# Healthy ageing and the prediction of mortality and incidence dependence in low- and middle- income countries: a 10/66 population-based cohort study

**DOI:** 10.1186/s12874-019-0850-5

**Published:** 2019-12-05

**Authors:** Christina Daskalopoulou, Martin Prince, Artemis Koukounari, Josep Maria Haro, Demosthenes B. Panagiotakos, A. Matthew Prina

**Affiliations:** 1 Department of Health Service and Population Research, King’s College London, Institute of Psychiatry, Psychology and Neuroscience, David Goldberg Centre, De Crespigny Park, London, SE5 8AF UK; 2 Department of Infectious Disease Epidemiology, London School of Hygiene & Tropical Medicine, Faculty of Epidemiology and Population Health, London, WC1E 7HT UK; 3 Parc Sanitari Sant Joan de Déu, Fundació Sant Joan de Déu, CIBERSAM, Universitat de Barcelona, Barcelona, Spain; 4 Department of Nutrition and Dietetics, School of Health Science and Education, Harokopio University, Athens, Greece

**Keywords:** Healthy ageing, Mortality, Incident dependence, 10/66, Functional ability

## Abstract

**Background:**

In the absence of a consensus on definition and measurement of healthy ageing, we created a healthy ageing index tallying with the functional ability framework provided by the World Health Organization. To create this index, we employed items of functional ability and intrinsic capacity. The current study aims to establish the predictive validity and discrimination properties of this healthy ageing index in settings in Latin American, part of the 10/66 cohort.

**Methods:**

Population-based cohort studies including 12,865 people ≥65 years old in catchment areas of Cuba, Dominican Republic, Venezuela, Mexico and Peru. We employed latent variable modelling to estimate the healthy ageing scores of each participant. We grouped participants according to the quintiles of the healthy ageing score distribution. Cox’s proportional hazard models for mortality and sub-hazard (competing risks) models for incident dependence (i.e. needing care) were calculated per area after a median of 3.9 years and 3.7 years, respectively. Results were pooled together via fixed-effects meta-analysis. Our findings were compared with those obtained from self-rated health.

**Results:**

Participants with lowest levels, compared to participants with highest level of healthy ageing, had increased risk of mortality and incident dependence, even after adjusting for sociodemographic and health conditions (HR: 3.25, 95%CI: 2.63–4.02; sub-HR: 5.21, 95%CI: 4.02–6.75). Healthy ageing scores compared to self-rated health had higher population attributable fractions (PAFs) for mortality (43.6% vs 19.3%) and incident dependence (58.6% vs 17.0%), and better discriminative power (Harrell’s c-statistic: mortality 0.74 vs 0.72; incident dependence 0.76 vs 0.70).

**Conclusion:**

These results provide evidence that our healthy ageing index could be a valuable tool for prevention strategies as it demonstrated predictive and discriminative properties. Further research in other cultural settings will assist moving from a theoretical conceptualisation of healthy ageing to a more practical one.

## Background

The number of people aged 60 years old and over will grow by 56% between 2015 and 2030; Latin American countries are expected to experience the fastest increase [1]. However, an increased life expectancy does not entail that these additional years will be spent in good health [2]. Old age has been associated with an increasing demand for care, services and expertise to treat or prevent the onset of non-communicable and chronic diseases [1]. In addition, the association between chronological age and health status is characterised by variability as there are differences in the health and functional status of older people [3]. People age with great diversity meaning that they could reach old age by remaining robust, dependent or in between [4]. Therefore, in this heterogeneous context, having a tool to identify those who are at greater risk of mortality or needing care will contribute to the establishment of effective policy interventions.

Research in healthy ageing, until recently, has been complicated by the lack of a consensus around its definition or measurement [5, 6]. During the last few years, a growing body of research has been shifted towards the creation of a healthy/successful ageing index and how the latter can assist in the prediction of health-related outcomes or mortality [7–9]. Indexes have been created either by using solely clinical biomarkers, [8] or a mixture of physiological, and psychosocial components [7, 9]. In response to the latest report World Health Organization (WHO) report [4] and to the “Global strategy and action plan on ageing and health (2016-2020)” [10], we created a healthy ageing index conceptualised upon the framework of functional ability (BMRM-D-18-00329). This index was developed in a population cohort of older adults from Latin American countries (Cuba, Dominican Republic, Mexico, Peru, Puerto Rico, Venezuela), part of the baseline survey of the 10/66 cohort. The index was checked for various psychometric properties, including measurement invariance among countries and gender, and concurrent convergent validity with self-rated health (BMRM-D-18-00329). In the current study, we aimed to investigate the predictive validity and the discrimination properties of this index on mortality and incident dependence risk in the follow-up wave of the 10/66 cohort. We also compare our findings with those of a measure of self-rated health, as evidence suggested that the latter has strong associations with mortality [11, 12] and with other adverse health outcomes [13, 14].

## Methods

### Design and setting of the study

The 10/66 Dementia Research Group (10/66 DRG) is a multicentre population-based study of ageing and dementia in low-and-middle income countries. Residents aged 65 years old and over were interviewed in the baseline surveys which took place between 2003 and 2010; follow-up surveys followed 3 to 5 years later. In cases where the participant could not be traced, information about vital status and current residence was sought from friends or family members, whose contract details had been recorded at baseline. In cases where participant’s capacity to provide reliable information was in doubt, the information was corroborated by an informant. The current secondary analyses included data from catchment areas in Latin America (Cuba, Dominican Republic, Peru, Venezuela, Mexico and Puerto Rico). Local ethical committees and the ethical committee of the Institute of Psychiatry of King’s College London approved the studies [15, 16]. In this study, we will describe aspects directly relevant to the presented analyses.

### Healthy ageing index

The healthy ageing index comprised 26 health question-indicators which were either self-reported or provided by key informants from the following questionnaires and questions.

- difficulty in: household responsibilities, walking a kilometre, washing whole body, getting dressed, carrying out work and everyday activities (World Health Organization Disability Assessment Schedule 2.0, WHO-DAS II)
- difficulty in: making decisions, using the toilet, handling money, finding right word, completing chores; change in daily activities and forgets where he/she is (Community Screening Interview for Dementia-Informant Scale, CSI’D’-RELSCORE)
- sleep trouble or recent change in pattern; feeling of not coping properly with everyday routine; getting worn out or exhausted during daytime or evening (Geriatric Mental State Interview, GMS)
- delayed recall; long memory test; immediate recall; verbal fluency; time orientation; praxis-fold a piece of paper; story recall difficulty (Neurological Examination and Community Screening Interview for Dementia, CSI’D’)
- word list learning; time in seconds taken to walk 10 m; hearing problem; sight problem. All interview manuals and questionnaires are available, upon request, on the official site of the study: https://www.alz.co.uk/1066/population_based_study_prevalence.php.

The structural validity of this index in the 10/66 cohort together with psychometric properties and measurement invariance tests among countries and between men and women have been previously investigated (BMRM-D-18-00329) [17]. Within that study, a bifactor model emerged as the most appropriate one to conceptualise the latent construct of healthy ageing. More specifically, healthy ageing was portrayed by the general factor within the bifactor model framework, whereas four other subdomain factors captured additional variability. For the purposes of the following analyses, we extracted the factor scores of the general factor using Mplus 7.4 [18]. Finally, we transformed scores in a 0 to 100 scale with higher values indicating participants in a better level of health.

### Mortality outcome

Mortality was ascertained through screening of all respondents from the baseline cohort. A verbal autopsy on those deceased and a suitable informant interview were completed to ascertain cause of death. Survival times were calculated up to the date of death or were censored to the date of follow-up survey for those participants who were re-interviewed. For those who refused interview but were found alive the median date of follow-up interview was used to calculate censor time.

### Incident dependence outcome

The need for frequent help that a healthy adult requires has been described as dependence [19]. In the 10/66 dataset, dependence was coded in three categories: no need for care, care some of the time and care much of the time [20]. The interviewer assigned each participant to these categories after asking the following questions to an informant: who shares the home with the participant? what kind of help does the participant need inside and outside the house? who, in the family, is available to care for the participant? what help do you provide? do you help to organise care and support for the participant? is there anyone else in the family more involved in helping than you and what do they do? what about friends and neighbours, what do they do? [21]. The same approach was used in the baseline and follow-up surveys. Participants with no need for care at baseline were at risk for the onset of dependence in the follow-up survey; participants who were rated as needing care some or much of the time were considered as having incident dependence. Incident dependence for deceased participants before death was extracted after informants’ interviews. “Time-at-risk” was calculated as the total follow-up time (until follow-up interview or death); for those participants that had a dependence event it was assumed that the onset of dependence occurred at the midpoint of the follow-up period.

### Covariates

We recorded participants’ age, sex, educational level (none, did not complete primary, completed primary, secondary or tertiary) and number of assets out of the following seven: car, television, refrigerator, telephone, mains water, mains electricity, plumbed toilet. We also measured and adjusted our models for physical impairments, stroke, dementia and depression as previous studies showed that these are considerable contributors of dependence and disability [22, 23]. The number of self-reported limiting physical impairments was assessed by the following list: arthritis or rheumatism, persistent cough, breathlessness, difficulty breathing or asthma, high blood pressure, heart trouble or angina, stomach or intestine problems, faints or blackouts, paralysis, weakness or loss of one leg or arm, skin disorders such as pressure sores, leg ulcers or severe burns. We then categorised participants as having: no, one to two and three or more illnesses. Self-reported stroke was confirmed if the interviewer or the informant confirmed that the former had characteristic symptoms lasting more than 24 h [24]. Depression was assessed using ICD10 (International Classification of Diseases) criteria and dementia was diagnosed by the cross-culturally developed, calibrated and validated 10/66 dementia diagnosis algorithm [25] and the Diagnostic and Statistical Manual of Mental Disorders (DSM-IV) manual [26]. For the needs of the current study, we separately provide the baseline characteristics for those at risk of mortality (mortality cohort) and for those with no need for care at baseline but for risk of incident dependence later (dependence cohort).

## Statistical analyses

We performed all statistical analyses in STATA 14.1 [27]. We used cohort-specific quintiles of the healthy ageing factors scores and used them as cut-off points to categorise participants in five groups of healthy ageing. Q1-quintile participants were those with the lowest level of healthy ageing “very low level” group in the baseline assessment, whereas Q5-quintile participants were those of the highest level “very high level” group. Q2-quintile participants were characterised as “low level”, Q3 as “moderate level” and Q4 as “high level”.^1^ Participants belonging to the highest level were used as reference category for all analyses.

We examined unadjusted Kaplan-Meier curves and employed a log-rank test to statistically test whether there are differences in participants’ death rate among the different healthy ageing levels. We constructed univariable and multivariable Cox’s proportional hazard (PH) models to estimate hazard ratios (HR) with 95% CIs and associations of mortality with healthy ageing in each country. Cox’s PH models were implemented on no missing data (< 2.5% of the data were excluded due to missingness in the covariates). We tested the PH assumption by inspecting the Kaplan-Meier curves together with the Schoenfeld residuals global test [28].

We performed competing risk analysis to quantify the risk of incident dependence by considering those participants who had the competing risk of dependence free death. Participants, for the competing risk analysis, were characterised as:

- Censored: survived and participated in the follow-up survey with no need for care.
- Incident dependent: identified as dependent from the follow-up interview or, if dead, from the informant deceased interview.
- Having competing event: dead but with no incident dependence as identified by the informant deceased interview.

We modelled the effect of covariates on incident dependence with a competing-risks regression derived from Fine and Gray’s proportional sub-hazards model [29]. This model (STATA stcrreg command) is based on a cumulative incidence function, indicating the probability of failure (i.e. onset of dependence) before a specific time but by considering the possibility of another competing risk (i.e. dependence-free death). In a conventional Cox’s PH regression, deaths would be right-censored, and those participants would be treated as no more or less likely to fail from the cause of interest than participants still at risk. However, this type of censoring is inappropriate as after death, dependence is a non-possible event. On the contrary, in the competing risk regression model these participants with a competing risk event are included in the analysis and are counted as having no chance of failing the event of interest [28]. The sub-hazard ratios (sHR) reflect the incident dependence ratio among participants who were alive at the end of the follow-up or experienced a death event free of incident dependence.

Cox’s PH models and sub-HRs models were calculated with robust 95%CIs considering household clustering. To combine and provide a pooled result among countries, we applied fixed-effects meta-analysis. We computed Higgins I^2^ to estimate the proportion of between-site variability in estimates accounted for heterogeneity, rather than sampling error [30]. Values of 50% are usually considered as moderate heterogeneity and values of 75% are considered as high [30]. We also calculated the population attributable fraction (PAF) with 95%CIs for mortality and incident dependence of a dichotomised healthy ageing exposure in the sociodemographic-adjusted model (STATA punaf command). This command estimates the attributable fractions under two different scenarios for the exposure variable (i.e. healthy ageing level). PAFs represent the proportion of mortality and incident dependence that could theoretically be avoided if all exposure could be removed from the population, assuming causal relationships estimated free of confounding. Furthermore, we calculated Harrell’s c-statistic to assess discrimination properties of the healthy ageing index for death and dependence events (STATA somersd command) [31]. Discrimination quantifies the ability of a model to correctly classify participants within the different groups of the outcome variable; a value of 0.5 indicates no predictive discrimination, whereas a value of 1.0 indicates perfect separation [32]. We also compared results of associated risks with regards to the outcomes of mortality and incident dependence by using as exposure the self-rated health of participants in the past 30 days instead of the healthy ageing. Finally, we performed a sensitivity analysis to examine the practical usefulness of this healthy ageing. We investigated if our conclusions would be affected, if a simple sum of items, and not the healthy ageing scores of the latent variable model, was employed.

## Results

### Sample study characteristics

Baseline characteristics of the mortality and dependence free cohorts are provided per country and in aggregate form (Tables 1 and 2). The mortality cohort comprised 12,734 individuals at baseline and for most of them (87.9%) their vital status was available in the follow-up wave. Mortality rates ranged from 28.4/1000 person-years (Peru) to 62.7/1000 person-years (Dominican Republic). The dependence cohort comprised 11,040 individuals with no need for care at baseline and more than 80% of those had their status ascertained in the follow-up wave. Incident dependence ranged from 33.4/1000 person-years (Cuba) to 64.8/1000 person-years (Dominican Republic). Venezuela and Peru had the highest number of participants with “very high level” of healthy ageing while Dominican Republic and Mexico had the lowest. The mean age ranged from 72.5 in Venezuela to 76.3 in Puerto Rico for the mortality cohort and from 71.5 in Venezuela to 75.2 in Puerto Rico for the incident dependence cohort. The majority of participants in both cohorts were females (> 63%), with six assets (median number) and with no limiting impairments or diseases (i.e. stroke, depression, dementia).

**Table 1:** Mortality cohort characteristics

Mortality Cohort	Cuba	Dominican Republic	Mexico	Peru	Puerto Rico	Venezuela	Total
Baseline sample (alive at baseline)	2813	2011	2003	1933	2009	1965	12,734
Vital status determined (% of baseline)	2635 (93.7%)	1706 (84.8%)	1844 (92.1%)	1752 (90.6%)	1563 (77.8%)	1697 (86.4%)	11,197 (87.9%)
Interviewed (% of baseline sample)	2007 (71.3%)	1197 (59.5%)	1459 (72.8%)	1311 (67.8%)	1265 (63.0%)	1257 (64.0%)	8496 (66.7%)
Deaths (% of those with vital status determined)	608 (23.1%)	467 (27.4%)	209 (11.3%)	152 (8.7%)	298 (19.1%)	200 (11.8%)	1934 (17.3%)
Person years of follow-up	10,844.6	7448.6	5366.7	5356.8	6474.3	7031.0	42,521.9
Mortality rate per 1000 person-years	56.1	62.7	38.9	28.4	46.0	28.4	45.5
Median years of follow-up (25th and 75th centile)	4.2 (3.5–5.0)	5.0 (3.6–5.1)	3.0 (2.9–3.1)	3.0 (2.5–3.7)	4.3 (3.7–4.7)	(4.0–4.8)	3.9 (3.0–4.8)
Mean age at baseline (SD)	75.2 (7.1)	75.3 (7.5)	74.3 (6.7)	74.8 (7.4)	76.3 (7.4)	72.5 (6.9)	74.8 (7.2)
Female Sex (%)	1836 (65.3%)	1325 (65.9%)	1268 (63.3%)	1183 (61.2%)	1347 (67.0%)	1252 (63.7%)	8211 (64.5%)
None did not complete primary education (%)	692 (24.6%)	1414 (70.3%)	1418 (70.8%)	352 (18.2%)	461 (22.9%)	601 (30.6%)	4938 (38.8%)
Median number of assets (25th–75th centile)	6.0 (5.0–6.0)	5.0 (4.0–6.0)	6.0 (4.0–6.0)	6.0 (6.0–6.0)	7.0 (6.0–7.0)	6.0 (6.0–7.0)	6.0 (5.0–7.0)
No limiting physical impairment (%)	1207 (42.9%)	599 (29.8%)	835 (41.7%)	887 (45.9%)	708 (35.2%)	748 (38.1%)	4984 (39.1%)
No stroke (%)	2605 (92.6%)	1842 (91.6%)	1871 (93.4%)	1825 (94.4%)	1841 (91.6%)	1846 (93.9%)	11,830 (92.9%)
No ICD10 depressive episode (%)	2671 (95.0%)	1733 (86.2%)	1911 (95.4%)	1830 (94.7%)	1962 (97.7%)	1858 (94.6%)	11,965 (94.0%)
No dementia (%)	2517 (89.5%)	1769 (88.0%)	1823 (91.0%)	1767 (91.4%)	1765 (87.9%)	1820 (92.6%)	11,461 (90.0%)
Q1 of healthy ageing (highest level)	660 (23.5%)	199 (9.9%)	274 (13.7%)	482 (24.9%)	347 (17.3%)	576 (29.3%)	2538 (19.9%)
Self-rated health: very good	287 (10.2%)	272 (13.5%)	392 (19.6%)	409 (21.2%)	157 (7.8%)	288 (14.7%)	1805 (14.2%)

*SD* standard deviation, *ICD* International Classification of Diseases

**Table 2:** Dependence cohort characteristics

Dependence Cohort	Cuba	Dominican Republic	Mexico	Peru	Puerto Rico	Venezuela	Total
Baseline sample (no needs for care at baseline)	2225	1770	1807	1770	1714	1754	11,040
*Follow-up tracing*							
Interviewed (% of baseline sample)	1679 (75.5%)	1103 (62.3%)	1352 (74.8%)	1234 (69.7%)	1174 (68.5%)	1154 (65.8%)	7696 (69.7%)
Deceased (% of baseline sample)	386 (17.3%)	345 (19.5%)	159 (8.8%)	97 (5.5%)	176 (10.3%)	139 (7.9%)	1302 (11.8%)
Refused (% of baseline sample)	12 (0.5%)	39 (2.2%)	164 (9.1%)	273 (15.4%)	5 (0.3%)	217 (12.4%)	710 (6.4%)
Lost (% of baseline sample)	148 (6.7%)	283 (16.0%)	132 (7.3%)	166 (9.4%)	359 (20.9%)	244 (13.9%)	1332 (12.1%)
Participants contribute to competing risk analysis (% of baseline sample)	2065 (92.8%)	1448 (81.8%)	1511 (83.6%)	1331 (75.2%)	1350 (78.8%)	1293 (73.7%)	8998 (81.5%)
Incidence dependence^a^	271 (13.1%)	376 (26.0%)	209 (13.8%)	156 (11.7%)	276 (20.4%)	238 (18.4%)	1526 (17.0%)
Competing risk (dependence free death)	78 (3.8%)	202 (14.0%)	66 (4.4%)	55 (4.1%)	116 (8.6%)	80 (6.2%)	597 (6.6%)
Person-years at risk for competing risk analysis (divided by two for incident dependence)	8106.6	5800.9	4132.9	3928.2	5196	4944.1	32,108.7
Incidence rate per 1000 person-years	33.4	64.8	50.6	39.7	53.1	48.1	47.5
Median person-years at risk for competing risk analysis (25th and 75th centile)	4.1 (3.2–4.9)	4.9 (2.5–5.1)	3.0 (2.8–3.1)	3.0 (2.4–3.7)	4.2 (2.9–4.6)	4.2 (3.5–4.6)	3.7 (2.7–4.6)
For those contributing in competing risk analysis							
Mean age at baseline (SD)	74.4 (6.6)	74.5 (7.0)	73.9 (6.4)	74.3 (7.1)	75.2 (6.5)	71.5 (6.1)	74.0 (6.7)
Female Sex (%)	1296 (62.8%)	949 (65.5%)	957 (63.3%)	802 (60.3%)	903 (66.9%)	808 (62.5%)	5715 (63.5%)
None or did not complete primary education (%)	479 (23.2%)	1028 (71.0%)	1051 (69.6%)	256 (19.2%)	265 (19.6%)	363 (28.1%)	3442 (38.3%)
Median number of assets (25th–75th centile)	6.0 (5.0–6.0)	5.0 (4.0–6.0)	6.0 (4.0–6.0)	6.0 (5.0–6.0)	7.0 (7.0–7.0)	6.0 (6.0–7.0)	6.0 (5.0–7.0)
No limiting physical impairment (%)	918 (44.5%)	448 (30.9%)	654 (43.3%)	614 (46.1%)	517 (38.3%)	520 (40.2%)	3671 (40.8%)
No stroke (%)	1945 (94.2%)	1347 (93.0%)	1432 (94.8%)	1279 (96.1%)	1271 (94.1%)	1231 (95.2%)	8505 (94.5%)
No ICD10 depressive episode (%)	1993 (96.5%)	1269 (87.6%)	1456 (96.4%)	1273 (95.6%)	1325 (98.1%)	1241 (96.0%)	8557 (95.1%)
No dementia (%)	1969 (95.4%)	1323 (91.4%)	1426 (94.4%)	1270 (95.4%)	1299 (96.2%)	1259 (97.4%)	8546 (95.0%)
Q1 of healthy ageing (highest level)	477 (23.1%)	148 (10.2%)	196 (13.0%)	324 (24.3%)	242 (17.9%)	409 (31.6%)	1796 (20.0%)
Self-rated health: very good	236 (11.4%)	207 (14.3%)	299 (19.8%)	284 (21.3%)	116 (8.6%)	215 (16.6%)	1357 (15.1%)

*SD* standard deviation, *ICD* International Classification of Diseases, ^a^either identified at follow-up interview or predicted from the informant deceased interview

### Associations between healthy ageing and mortality

**Fig. 1:**
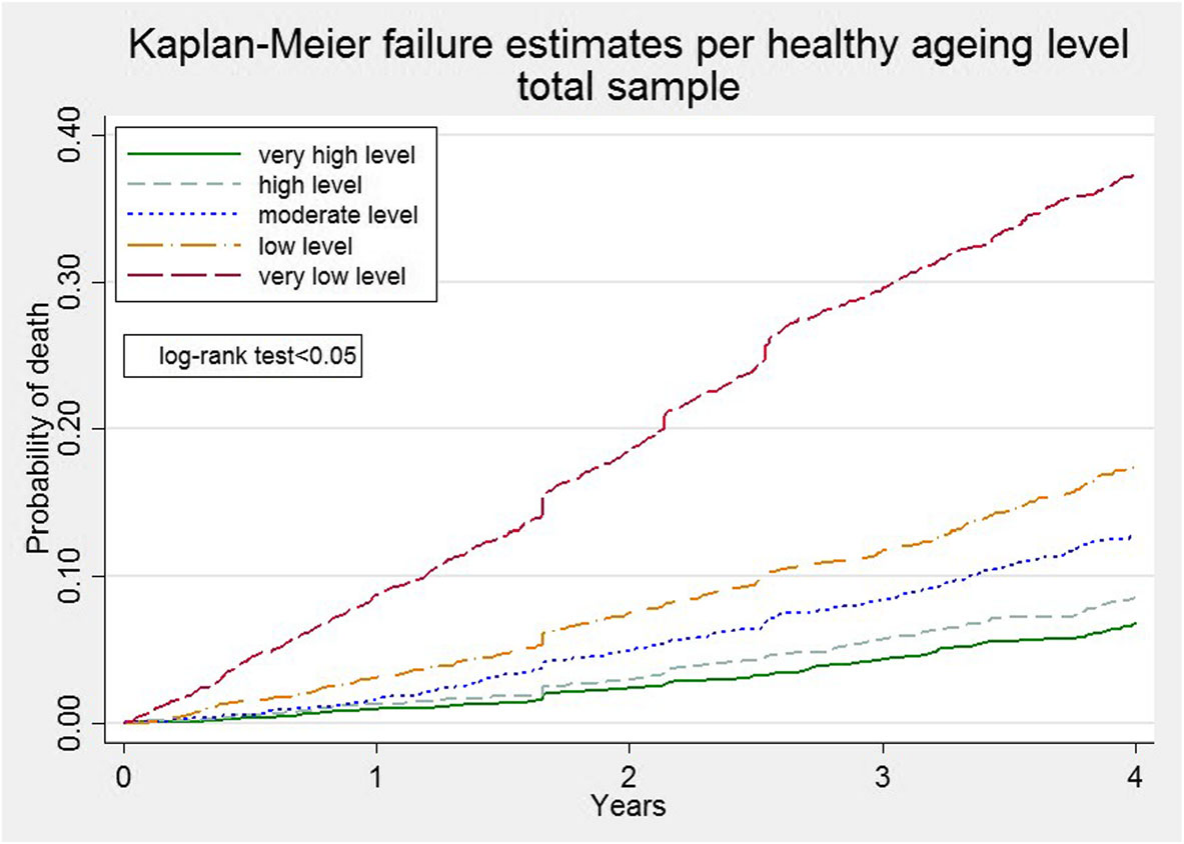
Kaplan-Meier failure estimates per healthy ageing level

Kaplan-Meier failure (death) curves per healthy ageing level are presented in Fig. 1. Log-rank test indicated a significant difference in time to death among the five levels of healthy ageing (*p* < 0.05). The Kaplan-Meier failure probability estimates at four years were about 0.35 for “very low level” participants, 0.18 for “low level”, 0.12 for “moderate level” and less than 0.10 for “high level” and “very high level”.

The Cox’s PH models for the healthy ageing index and self-rated health are presented in Table 3. The Schoenfeld residuals global test indicated that in most of the cases the proportional hazard assumption was met (*p*-value> 0.05). In one case where the global test indicated a significant result (in Puerto Rico for the fully adjusted models), further inspection of the Kaplan-Meier curves provided support that substantial deviations of the proportional hazard assumption were not detected. Table 3 shows that in the unadjusted model (model 1), “very low level” participants had an increased risk of death compared to “very high level” participants (HR = 6.89, 95%CI = 5.81–8.17) with moderate heterogeneity among countries (I^2^ = 53.40%). When we adjusted this model for sociodemographic characteristics (model 2), the association remained strong with less heterogeneity of effect among areas (HR = 4.23, 95%CI = 3.48–5.13; I^2^ = 17.10%). After further controlling for health conditions (model 3), associations were attenuated but remained significant (HR = 3.25, 95%CI = 2.63–4.02; I^2^ = 28.70%). Participants with “low” and “moderate” healthy ageing levels compared with participants of “very high” level had increased HR in all three models; effect sizes were progressively reduced after sequentially controlling for sociodemographic characteristics and health conditions but remained significant. “High level” participants compared to “very high level” had increased risk of mortality in the unadjusted model which did not remain significant once we adjusted for the other covariates. Regarding self-rated health and mortality, participants with very low and low self-rated health had an increased risk of mortality even in the fully-adjusted models compared to those reporting very good self-rated health (very low: HR = 2.78, 95%CI = 2.05–3.76; low: HR = 1.68, 95%CI = 1.35–2.09). Participants of moderate and good self-rated health had increased risk of death which did not remain significant as we subsequently adjusted for more covariates.

**Table 3:** Meta-analysed effects of healthy ageing and self-rated health on mortality, controlling sequentially for sociodemographic and health conditions

Hazard Ratios (HR) and 95%CI					
Healthy Ageing	Q1 quintile	Q2 quintile	Q3 quintile	Q4 quintile	Q5 quintile
Model 1	6.89 (5.81–8.17)	2.61 (2.16–3.14)	1.89 (1.55–2.29)	1.26 (1.02–1.56)	reference
	I^2^ = 53.4%, *p* = 0.057	I^2^ = 0.0%, *p* = 0.431	I^2^ = 0.0%, *p* = 0.615	I^2^ = 29.8%, *p* = 0.212	
Model 2	4.23 (3.48–5.13)	2.03 (1.67–2.48)	1.60 (1.31–1.95)	1.16 (0.94–1.44)	reference
	I^2^ = 17.1%, *p* = 0.304	I^2^ = 0.0%, *p* = 0.585	I^2^ = 0.0%, *p* = 0.714	I^2^ = 22.6%, *p* = 0.264	
Model 3	3.25 (2.63–4.02)	1.88 (1.53–2.30)	1.56 (1.27–1.91)	1.15 (0.93–1.43)	reference
	I^2^ = 28.7%, *p* = 0.220	I^2^ = 0.0%, *p* = 0.524	I^2^ = 0.0%, *p* = 0.726	I^2^ = 26.0%, *p* = 0.239	
Self-rated health	very low	low	moderate	good	very good
Model 1	4.65 (3.53–6.13)	2.36 (1.94–2.88)	1.33 (1.14–1.55)	1.06 (0.91–1.25)	reference
	I^2^ = 68.0%, *p* = 0.008	I^2^ = 73.5%, *p* = 0.002	I^2^ = 30.4%, *p* = 0.207	I^2^ = 57.0%, *p* = 0.040	
Model 2	4.12 (3.07–5.54)	2.09 (1.71–2.57)	1.24 (1.06–1.45)	0.99 (0.85–1.17)	reference
	I^2^ = 64.6%, *p* = 0.015	I^2^ = 74.5%, *p* = 0.001	I^2^ = 61.7%, *p* = 0.023	I^2^ = 67.6%, *p* = 0.009	
Model 3	2.78 (2.05–3.76)	1.68 (1.35–2.09)	1.16 (0.98–1.36)	0.98 (0.83–1.15)	reference
	I^2^ = 55.0%, *p* = 0.049	I^2^ = 73.9%, p = 0.002	I^2^ = 51.3%, *p* = 0.068	I^2^ = 69.9%, *p* = 0.005	

*HR* hazard rate, CI confidence intervals, Q1: lowest level of healthy ageing; Q5: highest level of healthy ageing; Model 1: no adjustments; Model 2: adjusted for age, sex, education level, number of assets; Model 3: Model 2 + physical impairments, stroke, depression, dementia

The model with only including healthy ageing scores (i.e. unadjusted model) had better discrimination for death compared to the model only including self-rated health (c-statistic: 0.70 vs 0.58, *p* < 0.05). Healthy ageing also predicted more strongly mortality, compared to self-rated health, even after sociodemographic adjustment (c-statistic: 0.74 vs 0.72, *p* < 0.05). Finally, addition of the healthy ageing index significantly improved the discrimination for death in a model already adjusted for age, sex, education and number of assets (c-statistic: mortality: 0.74 vs 0.70, *p* < 0.05).

### Associations between healthy ageing and incident dependence

Table 4 shows the pooled meta-analysed sub-hazard ratios (sHR) for the association of healthy ageing and self-rated health with incident dependence. Regarding healthy ageing, the sub-hazard risk was considerably higher for the “very low level” participants compared to “very high level” in all models (model 1: sHR = 10.61, 95%CI = 8.38–13.43; model 2: sHR = 7.76, 95%CI = 6.04–9.97; model 3: sHR = 5.21, 95%CI = 4.02–6.75). In addition, participants in all other levels had increased risk compared to “very high level” participants; strength of association, in most of the cases, was lessened when moving to higher levels and as we adjusted for more covariates. However, all associations remained significant with limited heterogeneity. Regarding self-rated health and incident dependence, participants with low and moderate self-rated health had increased sub-hazard risk even in the fully-adjusted models (low: sHR = 1.70, 95%CI = 1.29–2.24; moderate: sHR = 1.29, 95%CI = 1.07–1.55). Participants with very low self-rated health had increased risk compared to participants of very good self-rated health which did not remain significant once we adjusted for more covariates. Finally, participants with good self-rated health had no statistically different sub-hazard risk compared to very good self-rated health participants in all models.

**Table 4:** Meta-analysed effects of healthy ageing and self-rated health on incident dependence, controlling sequentially for sociodemographic and health conditions

Incident dependence sub-HR (95% CI)					
Healthy Ageing	Q1 quintile	Q2 quintile	Q3 quintile	Q4 quintile	Q5 quintile
Model 1	10.61 (8.38–13.43)	4.38 (3.42–5.61)	2.85 (2.20–3.68)	1.86 (1.42–2.45)	reference
	I^2^ = 0.0%, *p* = 0.468	I^2^ = 0.0%, *p* = 0.696	I^2^ = 0.0%, *p* = 0.663	0.0%, *p* = 0.670	
Model 2	7.76 (6.04–9.97)	3.63 (2.80–4.70)	2.52 (1.94–3.27)	1.72 (1.30–2.27)	reference
	I^2^ = 0.0%, *p* = 0.949	I^2^ = 0.0%, *p* = 0.915	I^2^ = 0.0%, *p* = 0.904	I^2^ = 0.0%, *p* = 0.830	
Model 3	5.21 (4.02–6.75)	3.53 (2.71–4.59)	2.60 (2.00–3.35)	1.74 (1.32–2.29)	reference
	I^2^ = 0.0%, *p* = 0.735	I^2^ = 0.0%, *p* = 0.819	I^2^ = 0.0%, *p* = 0.889	I^2^ = 0.0%, *p* = 0.799	
Self-rated health	very low	low	moderate	good	very good
Model 1	2.18 (1.30–3.64)	2.33 (1.84–2.94)	1.54 (1.30–1.83)	1.14 (0.96–1.35)	reference
	I^2^ = 47.4%, *p* = 0.107	I^2^ = 65.1%, *p* = 0.014	I^2^ = 54.6%, *p* = 0.051	I^2^ = 24.1%, *p* = 0.253	
Model 2	1.77 (0.99–3.18)	2.03 (1.60–2.57)	1.37 (1.15–1.62)	1.02 (0.85–1.22)	reference
	I^2^ = 1.3%, *p* = 0.399	I^2^ = 54.6%, p = 0.051	I^2^ = 59.2%, *p* = 0.031	I^2^ = 39.6%, *p* = 0.141	
Model 3	1.51 (0.77–2.94)	1.70 (1.29–2.24)	1.29 (1.07–1.55)	1.01 (0.84–1.21)	reference
	I^2^ = 0.0%, *p* = 0.529	I^2^ = 0.0%, *p* = 0.616	I^2^ = 19.7%, *p* = 0.285	I^2^ = 4.1%, *p* = 0.390	

*sub-HR* sub-hazard rates of competing risk analysis, *CI* confidence intervals, Q1: lowest level of healthy ageing; Q5: highest level of healthy ageing; Model 1: no adjustments; Model 2: adjusted for age, sex, education level, number of assets; Model 3: Model 2 + physical impairments, stroke, depression, dementia

Healthy ageing scores (unadjusted model) had better discrimination for the incident dependence risk compared to self-rated health (c-statistic: 0.72 vs 0.56, *p* < 0.05). Healthy ageing measure was a better predictor of incident dependence, compared to self-rated health, even after sociodemographic adjustment (c-statistic: 0.76 vs 0.70, *p* < 0.05). Finally, addition of the healthy ageing index significantly improved the discrimination for onset of dependence in the adjusted model (adjusted for age, sex, education and number of assets) (c-statistic: 0.76 vs 0.69, *p* < 0.05).

### Population attributable fraction

We compared PAFs of healthy ageing and self-rated health as dichotomous exposures (i.e. participants of “very high” and “high” healthy ageing level versus all other; participants of very good and good self-rated health versus all other) for their contribution to mortality and onset of dependence. For both outcomes, the contribution of the dichotomised healthy ageing exposure exceeded that of the dichotomised self-rated health exposure (Table 5). PAFs of healthy ageing scores among countries ranged from 32.4% (Mexico) to 52.5% (Peru) (weighted mean 43.6%) for death risk and from 51.3% (Venezuela) to 64.8% (Peru) (weighted mean 58.6%) for incident dependence. PAFs of self-rated health ranged from 9.4% (Dominican Republic) to 31.1% (Venezuela) (weighed mean 19.3%) for mortality and from 6.7% (Puerto Rico) to 29.8% (Venezuela) (weighted mean 17.0%) for incident dependence. This means that, by assuming residual free confounding, if we were able to have people in the two higher levels of healthy ageing only (Q4 and Q5 quintiles) then mortality would have been avoided by 43.6% and incident dependence by 58.6%. Accordingly, if all participants were of very good or good level of self-rated health, mortality and dependence would have been avoided by 18.4 and 17.0%, respectively.

**Table 5:** Population attributable fractions (PAFs) for the contribution of healthy ageing and self-rated health to mortality and incident dependence

Population attributable fraction (95% CI)		
	Healthy ageing	Self-rated health
	Mortality	
Cuba	44.0% (34.7–52.0%)	19.2% (11.3–26.5%)
Dominican Republic	33.6% (17.2–46.8%)	6.1% (−4.5–15.6%)
Mexico	32.4% (4.8–52.0%)	18.5% (4.6–30.4%)
Peru	52.5% (31.2–67.3%)	17.0% (−0.3–31.3%)
Puerto Rico	51.0% (34.5–63.3%)	19.8% (7.7–30.3%)
Venezuela	49.1% (33.7–60.9%)	30.0% (16.3–41.4%)
Weighted Mean	43.6%	18.4%
	Incident dependence	
Cuba	57.7% (44.5–67.7%)	16.8% (5.2–27.0%)
Dominican Republic	58.0% (43.8–68.6%)	7.4% (−3.6–17.3%)
Mexico	61.4% (40.4–75.0%)	18.3% (4.5–30.1%)
Peru	64.8% (44.6–77.6%)	24.6% (8.2–38.1%)
Puerto Rico	58.6% (45.8–68.4%)	6.7% (−5.2–17.3%)
Venezuela	51.3% (38.2–61.6%)	29.8% (18.1–39.9%)
Weighted Mean	58.6%	17.0%

*CI* Confidence Interval, Results are adjusted for age, sex, education level, number of assets; weighted mean is calculated by considering the number of participants in each country

### Sensitivity analysis

To test the practical usefulness of this healthy ageing measure, we also examined if a simple sum of the 26 items, and not factor scores, could be employed without meaningfully affect our conclusions. Cox’s models and competing risk analyses showed similar strength and direction of associations when sum of items was used as exposure (Additional file 1). Calculation of the relevant population attributable fractions also indicated overall good prediction of mortality and onset of dependence (but not better than the results where factor scores -and not simple sum of the 26 items- were considered).

## Discussion

In this study, we aimed to examine the predictive validity and the discrimination properties regarding mortality and incident dependence risk of a healthy ageing index constructed in the 10/66 cohort. We found that the healthy ageing index, tallying with the WHO framework of functional ability, can predict mortality and onset of dependence in the follow-up wave, even after adjusting for sociodemographic and health conditions. Relevant comparative analyses indicated that this measure had stronger associations with death risk and incident dependence compared to a measure of self-rated health. C-statistic also confirmed higher discrimination ability and calculation of PAFs showed that the healthy ageing scores provided a better overall prediction of mortality and incident dependent risk compared to self-rated health. Nevertheless, PAF calculations require the strong assumptions that the study is free of bias and that removing the exposure does not affect any other factors. The practical use of this index, as a population screening tool, was reinforced by the sensitivity analysis which revealed that a more simplistic version, easily used by health practitioners and care givers, can lead to similar conclusions.

Our findings are in accordance with those from other studies in which similar health metrics have been created. For instance, healthy ageing metrics conceptualised within the functional ability framework have been created in the English Longitudinal Study of Ageing (ELSA) [33] and the Health and Retirement Study (HRS) [34]. Both those metrics also showed good predictive ability against mortality and other adverse health outcomes (i.e. institutionalisation over 10 years). In addition, in comparison with another study in the same 10/66 populations, which examined the predictive ability of various frailty phenotypes, this healthy ageing index showed higher PAFs both for dependence and for mortality [20].

From our analyses, it is worth mentioning that participants in “high” or “very high” levels of healthy ageing did not differ in the prediction of mortality when adjusted for sociodemographic and health conditions. However, they did differ in the incident dependence risk. Future research could elucidate if these participants who did not differ in their mortality risk could be characterised as ‘survivors’ (living with an age-related disease diagnosed before old age), ‘delayers’ (living with an age-related disease diagnosed after their average life expectancy) or ‘escapers’ (living in old age without major disease) [35]. This finding reinforces even more the cruciality of ‘adding life to years as well as years to life’ mantra in policy interventions [4, 36, 37]. Considering, the heterogeneity in the ageing process, this index could potentially constitute a valuable tool in screening and identifying those who are at higher risk of incident dependence, but only after further testing and abbreviation.

Among the strengths of our study is the fact that to create this index respondents and key-informants’ data were employed and even individuals who were unable to provide information due to severe mental conditions or death were included in the sample. In agreement with the WHO report, which does not define healthy ageing by the absence or not of a disease [4], health conditions and diseases were not included in the conceptualisation of it. As a result, our healthy ageing tool provides great confidence that a broad spectrum of health was captured and that bias towards healthier participants was limited. In addition, our analyses were conducted on large population-based samples in Latin American countries, hence we were able to assess the consistency of the observed associations across different sites within this specific geographic area. Strength of associations did not differ among the different countries contributing to the generalisability of our findings. Furthermore, even though the study design was prospective and hence prone to attrition, modest attrition was observed which limited any potential information bias.

Limitations include that even though catchment areas were selected to be as representative as possible of the wider geographical region, our findings may not be generalisable beyond those specific areas. Cross-cultural differences in the conceptualisation of certain questions cannot be eliminated, even though interviewers underwent substantial training to ensure that questionnaires and procedures were implemented in the same way among countries. In addition, assessments were done in the baseline assessment and in people 65 years old and over. As a result, this healthy ageing index was not calculated in younger participants limiting the investigation of a life-course approach [38]. Future research should focus on the construction of this index in other longitudinal studies or in harmonised datasets of various cohorts. Building and validating indexes, which are composed by the same or similar items of functional ability, will assist in a better understanding of the ageing process and in the identification of the different ageing pathways that may exist.

## Conclusions

The results of our study support that this healthy ageing index, built within the functional ability framework of WHO, can predict mortality and incident dependence after a median of four years in subset of Latin America countries, part of the 10/66 population cohort. Given the rapid increase of older people’s population and the fact that the proportion of dependent people 60 years old and over is expected to almost double in between 2000 and 2050 in Latin America, [23] infrastructures for health and social care should be strengthened. An effective healthy ageing measure could be a valuable tool in developing and targeting effective primary and secondary prevention strategies.

## Supplementary information

**Additional file 1: Table S1.** Meta-analysed effects of healthy ageing as sum of items† on mortality and incident dependence, sequentially controlling for sociodemographic and health conditions. **Table S2.** Population attributable fractions (PAFs) for the contribution of healthy ageing as sum of items† to mortality and incident dependence.

## Data Availability

The 10/66 Dementia Research Group dataset is available upon request via the official site of the study: https://www.alz.co.uk/1066. All data generated in this study are available from the corresponding author on reasonable request.

## Abbreviations

CI: Confidence Intervals.
CSI’D’-RELSCORE: Community Screening Interview for Dementia-Informant Scale
df: degrees of freedom
DRG: Dementia Research Group
DSM-IV: Diagnostic and Statistical Manual of Mental Disorders IV
GMS: Geriatric Mental State Interview
HR: Hazard Ratio
ICD10: International Classification of Diseases 10th edition
PAF: Population Attributable Fraction
PH: Proportional Hazard
sHR: sub-Hazard Ratio
WHO: World Health Organization
WHO-DAS II: World Health Organization Disability Assessment Schedule 2.0

## Footnotes

1 Cut-off points of the mortality cohort: very low level: 0–47.80; low level: 48.81–58.13; moderate level: 58.14–66.74; high level: 66.75–78.08; very high level: 78.09–100. Cut-off points of the dependence cohort: very low level: 0–47.02; low level:47.03–56.53; moderate level: 56.54–65.12; high level: 65.13–73.83; very high level: 73.84–100.
