# Supplementary material for: Healthy ageing and the prediction of mortality and incidence dependence in low- and middle- income countries: a 10/66 population-based cohort study
Source: BMC Med Res Methodol. 2019 Dec 5;19:225. doi: 10.1186/s12874-019-0850-5 (PMC6894213; doi:10.1186/s12874-019-0850-5)
Supplement: Supplementary file 1 — Additional file 1: Table S1. Meta-analysed effects of healthy ageing as sum of items† on mortality and incident dependence, sequentially controlling for sociodemographic and health conditions. Table S2. Population attributable fractions (PAFs) for the contribution of healthy ageing as sum of items† to mortality and incident dependence. [file 12874_2019_850_MOESM1_ESM.docx]

**Supplementary File**

**Additional file 1: Table S1.** Meta-analysed effects of healthy ageing as sum of items† on mortality and incident dependence, sequentially controlling for sociodemographic and health conditions

| **Model** | **Q1 quintile** | **Q2 quintile** | **Q3 quintile** | **Q4 quintile** | **Q5 quintile** |
| --- | --- | --- | --- | --- | --- |
| **Hazard Ratios HR (95%CIs)** | | | | | |
| Model 1 | 5.26 (4.33-6.39)  I^2^=0.0%, p=0.780 | 2.38 (1.93-2.93)  I^2^=8.10%, p=0.365 | 1.63 (1.30-2.03)  I^2^=0.0%, p=0.944 | 1.27 (1.01-1.60)  I^2^=0.0%, p=0.719 | reference |
|  |  |  |  |  |  |
| Model 2 | 3.53 (2.83-4.41)  I^2^=0.0%, p=0.826 | 1.84 (1.47-2.31)  I^2^=0.0%, p=0.480 | 1.41 (1.12-1.78)  I^2^=0.0%, p=0.886 | 1.16 (0.92-1.47) | reference |
|  |  |  |  | I^2^=0.0%, p=0.767 |  |
| Model 3 | 3.13 (2.45-3.99)  I^2^=0.0%, p=0.730 | 1.83 (1.45-2.30)  I^2^=0.0%, p=0.512 | 1.43 (1.13-1.81)  I^2^=0.0%, p=0.849 | 1.17 (0.92-1.47) | reference |
|  |  |  |  | I^2^=0.0%, p=0.731 |  |
|  |  |  |  |  |  |
| **Incident dependence sub-HR (95% CIs)** | | | | | |
| Model 1 | 9.67 (7.55-12.39)  I^2^=19.7%, p=0.285 | 4.92 (3.81-6.35)  I^2^=0.0%, p=0.742 | 2.92 (2.23-3.82)  I^2^=0.0%, p=0.510 | 1.94 (1.48-2.54)  I^2^=0.0%, p=0.964 | reference |
| Model 2 | 6.64 (5.07-8.69)  I^2^=0.0%, p=0.667 | 3.86 (2.94-5.08)  I^2^=0.0%, p=0.785 | 2.56 (1.94-3.39)  I^2^=16.3%, p=0.309 | 1.75 (1.32-2.32)  I^2^=0.0%, p=0.863 | reference |
| Model 3 | 5.53 (4.13-7.41) | 3.89 (2.93-5.16) | 2.67 (2.02-3.54) | 1.83 (1.38-2.43) | reference |
|  | I^2^=0.0%, p=0.704 | I^2^=0.0%, p=0.859 | I^2^=0.0%, p=0.512 | I^2^=0.0%, p=0.908 |  |

sub-HR: sub-hazard rate; CIs: Confidence Intervals; Model 1: no adjustments; Model 2: adjusted for age, sex, education level, number of assets; Model 3: Model 2 + physical impairments, stroke, depression, dementia.

†Items were binary or ordinal variables; sum of items was calculated for each participant and cohort-specific quintiles were created. Participants belonging to the 1^st^, 2^nd^, 3^rd^, and 4^th^ quintile of the sum of items were compared to participants of the 5^th^ quintile (higher quintiles represent better level of healthy ageing).

Supplementary Table 2: Population attributable fractions (PAFs) for the contribution of healthy ageing as sum of items† to mortality and incident dependence

| **Population attributable fraction (95% CIs) for items count** | | |
| --- | --- | --- |
| **Country** | **Mortality** | **Incident dependence** |
| Cuba | 41.8% (30.9% - 50.9%) | 58.5% (45.0% - 68.6%) |
| Dominican Republic | 39.1% (22.0% - 52.4%) | 58.1% (43.7% - 68.8%) |
| Mexico | 25.7% (-1.8% - 45.7%) | 45.6% (23.9% - 61.1%) |
| Peru | 35.5% (7.3% - 55.1%) | 63.2% (43.0% - 76.2%) |
| Puerto Rico | 36.7% (15.1% - 52.7%) | 53.9% (40.5% - 64.3%) |
| Venezuela | 38.2% (17.5% - 53.8%) | 47.2% (32.5% - 58.7%) |
| **Weighted Mean** | **36.5%** | **55.6%** |

Results are adjusted for age, sex, education level, number of assets; weighted mean is calculated by considering the number of participants in each country. CIs: Confidence Intervals.

†Items were binary or ordinal variables; sum of items was calculated for each participant and cohort-specific quintiles were created. Participants belonging to the 1^st^, 2^nd^, 3^rd^, and 4^th^ quintile of the sum of items were compared to participants of the 5^th^ quintile (higher quintiles represent better level of healthy ageing).
